# Adrenocorticotropic hormone combined with vigabatrin as a second-line therapy for West syndrome

**DOI:** 10.1055/s-0045-1811172

**Published:** 2025-08-20

**Authors:** Luciana de Paula Souza, Danielle Caldas Bufara, Tallulah Spina Tensini, Sergio Antonio Antoniuk, Gustavo L. Franklin, Ana Chrystina de Souza Crippa

**Affiliations:** 1 Secretaria de Estado da Saúde, Departamento de Saúde do Paraná, Curitiba PR, Brazil.; 2 Universidade Federal do Paraná, Hospital de Clínicas, Unidade de Neuropediatria, Curitiba PR, Brazil.; 3 Universidade Federal do Paraná, Hospital de Clínicas, Departamento de Neurologia, Curitiba PR, Brazil.; 4 Universidade Federal do Paraná, Hospital de Clínicas, Departamento de Pediatria, Curitiba PR, Brazil.; 5 Pontifícia Universidade Católica do Paraná, Escola de Medicina, Departamento de Medicina Interna, Curitiba PR, Brazil.; 6 Universidade Federal do Paraná, Setor de Ciências da Saúde, Departamento de Clínica Médica, Curitiba PR, Brazil.

**Keywords:** Spasms, Infantile, Adrenocorticotropic Hormone, Vigabatrin

## Abstract

**Background:**

West syndrome is an epileptic encephalopathy for which combination therapies with adrenocorticotropic hormone and vigabatrin have emerged as new treatment options.

**Objective:**

To evaluate the clinical and electroencephalographic remission rates, tolerability, and relapse rates in patients with West syndrome who failed primary treatment and underwent sequential therapy with vigabatrin and adrenocorticotropic hormone.

**Methods:**

We included 39 patients with West syndrome from 2 specialized centers, aged 2 to 120 months. The patients were treated with intramuscular tetracosactide depot added to vigabatrin and were prospectively followed up for ≥ 1 year. The outcomes were clinical, and electroencephalographic remission rates at 7 and 30 days and 1 year following combined therapy initiation, progression to other epilepsy types, therapy tolerability, and relapse rates were recorded.

**Results:**

Of the original sample, 71% of the subjects were boys, and 87% had a known etiology. The clinical and electroencephalographic remission rates were 46.1%, 94.8% ( *p* = 0.001), and 74.1% ( *p* = 0.01) at 7 and 30 days, and 1 year after the initiation of combined therapy, respectively. At the 1-year follow-up, adverse effects were observed in 86.0% and the relapse rate was 21.6%. After a median follow-up of 21 months, 73.6% of the patients developed epilepsy.

**Conclusion:**

Combined therapy demonstrated a favorable efficacy profile in achieving clinical and electroencephalographic remission but was associated with significant seizure relapse rates in the medium term. Thus, it represents a feasible option for patients in whom initial treatment has failed.

## INTRODUCTION

West syndrome (WS) is characterized by epileptic spasms, hypsarrhythmia on electroencephalography (EEG), and neurodevelopmental delay or regression. 1 2 3 Patients with WS can present a challenging diagnosis and are often misdiagnosed with behavioral disorders, gastroesophageal reflux, or constipation, 4 which contributes to delayed treatment and worsened clinical outcomes. 2 3 4 5

The primary choices for WS therapy are adrenocorticotropic hormone (ACTH), prednisolone, and vigabatrin administration. 5 The superiority of hormonal treatment, provided mainly on an inpatient basis, has been reported, 6 despite a high treatment failure rate of approximately 30%. 7 New combination drug therapies provide superior efficacy but are also associated with high rates of adverse effects. 8

Initial data from patients with newly diagnosed WS 9 reveal that the presence of favorable prognostic factors improves the treatment response in the short-to-medium term. 10 Therefore, additional studies are needed to confirm the treatment effectiveness and tolerability, relapse rate, and progression to other neurological conditions in patients with primary treatment failure.

In the present study, we aimed to investigate the clinical and electroencephalographic remission (CER) and relapse rates of a combined therapy (CT) with vigabatrin and ACTH administration in the medium term by sequentially adding ACTH to vigabatrin in an outpatient setting. We hypothesized that this treatment protocol improves the CER rate and reduces the probability of progression to other epilepsy syndromes in patients with WS who did not respond to vigabatrin monotherapy.

## METHODS

### Study design

The study was conducted in two phases. First, an epileptologist reviewed the medical records of patients who underwent CT at two neuropediatric centers and retrieved data from January 2001 to June 2021 to determine CER rates and CT tolerability within the first 12 months after treatment initiation. In a second phase, patients were assessed during regular appointments or contacted for telephone interviews, after a minimum of one year of treatment to evaluate the relapse rate, CT tolerability, and progression rates to other epilepsy syndromes until the last follow-up.

### Definitions

West syndrome was defined as epileptic spasms associated with hypsarrhythmia on EEG and developmental delay or regression. 1 11 Conventional or video EEG was performed in the sleep, arousal, and awake states for at least 30 min each, and the results were interpreted by a neurophysiologist to investigate hypsarrhythmia. The new terminology infantile epileptic spasm syndrome (IESS) includes infants with WS and with epileptic spasms who do not fulfill all the criteria for WS. In our study, we specifically included individuals who met the criteria for WS. Therefore, we retained the term WS rather than using Infantile Epileptic Spasms Syndrome (IESS). 1 11

We defined CER as the absence of hypsarrhythmia patterns and epileptic spasms. Treatment failure was defined as the persistence of hypsarrhythmia or epileptic spasms despite the administration of a therapeutic dose of vigabatrin, with or without ACTH. The study defined relapse as the resurgence of clinical spasms and hypsarrhythmia after at least 30 days of CER. Patients without seizures for at least 6 months were considered seizure-free.

### Participants

Considering the low prevalence of WS, the study participants were enrolled via convenience sampling. The inclusion criteria were age between 2 months and 10 years; confirmed WS diagnosis; history of treatment failure despite optimized therapeutic doses of vigabatrin; subsequent treatment with both vigabatrin (> 100 mg/kg/day) and ACTH in an outpatient setting; and at least one follow-up visit one year after starting CT. The exclusion criteria were history of discontinuation of vigabatrin or ACTH during the treatment.

We retrospectively collected data on the patients' sex, age, age at spasm onset, underlying conditions associated with WS, gestational and neonatal history, neuroimaging abnormalities, treatment characteristics (such as time between diagnosis and initiation of vigabatrin or ACTH treatment), concomitant therapies (such as the use of other anti-seizure medications [ASMs]), and EEG findings. During CT and follow-up, we assessed the patients' ASM use, presence of epileptic spasms or hypsarrhythmia on EEG, other types of epileptic seizures, clinical complications, and therapy-related adverse effects.

### Outcomes

The primary outcome was WS resolution, defined by CER at days 7 and 30, and at 1 year after CT initiation. Secondary outcomes included the relapse rate, CT tolerability, and progression to other epilepsy syndromes at the last follow-up.

### Treatment protocol

The protocol to which they were subjected is a previously established institutional combined therapy protocol, adapted from a Brazilian study. 12 It utilized high ACTH doses based on previous reports suggesting improved long-term efficacy. 3 13 14 15 The WS diagnosis was confirmed in all patients through the observation of clinical spasms and hypsarrhythmia on conventional or video EEG, followed by magnetic resonance imaging (MRI), metabolic, and genetic investigations for underlying diseases. In patients who started WS treatment at other institutions and were not using vigabatrin or ACTH, vigabatrin was initiated, and its dose was adjusted as needed. In those already using vigabatrin, the appropriate dose optimization was performed, according to the following protocol to ensure the required therapeutic dose: vigabatrin was administered at 25 mg/kg per day, with the dose increased every 5 days until either CER was noted or the maximum tolerated dose (100–200 mg/kg per day) was reached. Once the epileptologist attested the treatment failure with vigabatrin, it was maintained at > 100 mg/kg per day, and ACTH was prescribed. Tetracosactide depot (Synacthen Depot, Novartis) was added to vigabatrin at a dose of 0.5 mg/kg per day and administered intramuscularly daily for up to 3 weeks. In patients with CER after 7 days of therapy, the ACTH and vigabatrin doses were not changed, and the patients completed 14 days of treatment. Non-responders continued receiving treatment for 21 days. For these individuals, the epileptologist changed the ACTH doses based on the percentage of hypsarrhythmias on EEG. Patients with hypsarrhythmias in more than 50% of EEG recordings received a double ACTH dose of 1 mg/kg per day for 2 additional weeks and received prednisolone for dose tapering. All other non-responders continued receiving the same dose (0.5 mg/kg per day) for 21 days. In cases of diagnostic uncertainty, the epileptologist used video EEG to confirm the WS diagnosis and assess the presence of CER at the 7-day, 30-day, and 1-year follow-ups.

### Statistical analyses

We used R version 4.2.0 (GUI) for the statistical analyses. The results are expressed as mean ± standard deviation, and median (interquartile range [IQRs]) values, or numbers and percentages. Ordinal and binary data were analyzed using Pearson's Chi-squared (χ ^2^ ) test and Fisher's exact test, respectively. McNemar's test was used to evaluate differences in categorical variables between groups according to the treatment response. Quantitative variables with a Gaussian distribution were compared using Student's t-test and the nonparametric Mann-Whitney test. Logistic regression analysis and forest plots were used to analyze the probabilities of the outcomes. To examine the time-dependent effects of treatment lag on therapy outcomes, we applied the Mann-Whitney test using the median, considering the nonparametric distribution of these data. We defined the level of significance as *p* < 0.05.

### Ethical statement

The current research was approved by the ethics committees of the 2 centers where the study was conducted (CAAE: 61921316.4.3002.0096 and 61921316.4.0000.0103). We confirm that we have read the Journal's position on issues involved in ethical publication and affirm that this work is consistent with those guidelines.

## RESULTS

**Figure 1:**
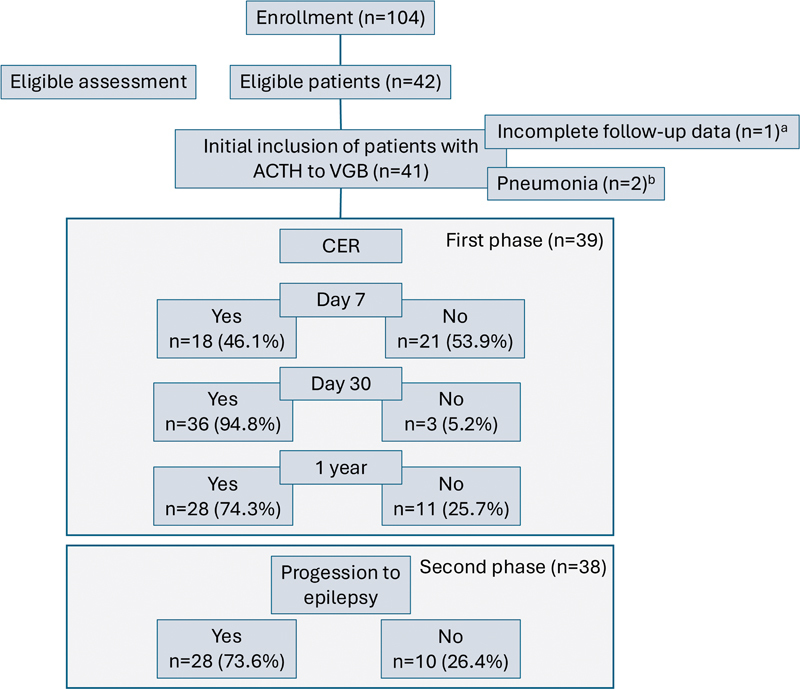
Abbreviations: ACTH, adrenocorticotropic hormone; CER, clinical and electroencephalographic remission; CT, combined therapy; VGB, vigabatrin. Notes: ^a^ Not eligible for enrollment. ^b^ These patients discontinued ACTH therapy due to infection and were excluded from the study. Schema showing participant enrollment and follow-up.

Among the 104 patients with WS treated at the 2 neuropediatric centers during the period, 39 were included in the retrospective phase, 38 of whom were included in the 2 ^nd^ phase ( Figure 1 ). The mean follow-up time was 37.8 ± 33.9 (range 12–144) months from the beginning of CT. In total, 28 patients (71.8%) were boys ( Table 1 ).

**Table 1:** Baseline characteristics of the patients

Characteristics (n = 39)	n (%)
Age at West syndrome diagnosis (months) ^a^	12 (7–18); 14 ± 13.3
Sex (male/female)	28/13
Mode of delivery	
Cesarean section	22 (56.4)
Gestational age (weeks)	36.3 ± 3.8
Birth weight (g) ^b^	2.714.0 ± 860.4
Apgar score at 1 min ≤ 3 ^c^	4 (10.5%)
Apgar score at 5 min ≤ 7 ^d^	4 (10.8%)
Neonatal injury	7 (17.9%)
Injury classification	
Prenatal factors	15 (38.5%)
Chromosomal abnormalities	11 (73.3%)
Malformations	4 (16.7%)
Perinatal factors	17 (43.6%)
Hypoxic-ischemic injury	7 (41.2%)
Prematurity	6 (35.3%)
Others	4 (23.6%)
Postnatal factors	2 (5.1%)
Family history of epilepsy	3 (7.7%)
Prior West syndrome diagnosis	15 (38.9%)
Other anti-seizure medications used	32 (82.1%)

Notes: ^a^ Data are presented as median and interquartile range (IQR) and mean ± standard deviation values; ^b^ n = 36; ^c^ n = 38; ^d^ n = 37.

In all the 39 participants, the onset of spasms occurred between 1 and 18 months of age (mean: 6 [IQR: 4–6] months). The etiology was known in 34 (87%) patients. Among them, 12 patients had no abnormalities on nuclear MRI. The most frequent structural abnormalities in the remaining 27 patients were leukomalacia (27%) and periventricular injuries (16.2%). Vigabatrin therapy was started at a median age of 5 (IQR 3–11) months, and ACTH therapy was added at a median age of 13 (IQR 9–19) months from the WS diagnosis.

**Figure 2:**
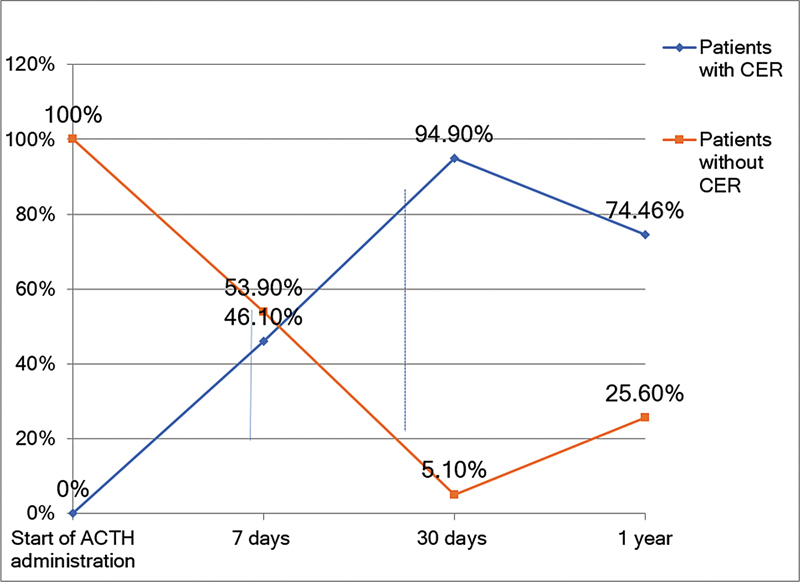
Abbreviations: ACTH, adrenocorticotropic hormone; CER, clinical and electroencephalographic remission (McNemar test). Clinical and electroencephalographic remission after sequential therapy.

Among the 39 initial patients, CER was achieved in 18 patients after 7 days (46.1%; *p* = 0.63); in 37 patients after 30 days (94.8%; *p* = 0.001); and in 29 patients after 1 year (74.3%; *p* = 0.01) of ACTH therapy ( Figure 2 ). Patients who initiated ACTH therapy sooner and at a younger age were more likely to achieve CER by the 7th day of treatment, although no statistically significant differences were observed ( *p* = 0.05 and *p* = 0.08, respectively). Premature patients were significantly more likely than term patients to maintain CER ( *p* = 0.04) until the 1-year follow-up. Clinical and electroencephalographic remission was not significantly associated with participant or treatment characteristics such as etiologies, and onset of spasms at any of the 3 time points ( *p* = 0.47, 0.10, and 0.48; 0.73, 0.57, and 0.65; and 1.0, 0.60, and 0.50 respectively) ( Table 2 ). Multivariate logistic regression analysis of data at 1 year of therapy revealed that female participants had an almost 5 times higher likelihood of an unfavorable outcome than male patients (odds ratio 5.52; 95% confidence interval 1.19–25.51; *p* = 0.05).

**Table 2:** Factors influencing clinical and electroencephalographic remission after 7 days, 30 days, and 1 year of treatment

Characteristics	CER: day 7	CER: day 30	CER: 1 year	*p*
Age at initiation of the ACTH therapy ^a^	11 (7–14)	15 (10–20)	12 (11–24)	0.08 ^e^
Vigabatrin dose (mg/kg per day) ^a^	150 (125–150)	136 (120–166)	150 (125–150)	> 0.05 ^e^
Time to ACTH therapy initiation (weeks) ^a*^	5 (2–10)	6 (3–14)	4 (3–11)	0.05 ^e^
Spasm onset ^a*^	5 (4–6, 7)	6 (4–8)	6 (4–7)	> 0.05 ^e^
Male sex	14 (77.7%)	14 (66.6%)	23 (79.3%)	> 0.05
Cesarean delivery	10 (55.5%)	12 (57.1%)	5 (45.4%)	> 0.05 ^f^
Prematurity	3 (16.7%)	6 (28.6%)	**9 (31.0%)**	**0.04** ^f^
Chromosomal abnormalities	3 (16.6%)	10 (27.0%)	7 (24.1%)	> 0.05 ^e^
Gestational age (weeks) ^a*^	38 (35–38)	38 (35–38)	38 (37.5–39.0)	> 0.05 ^e^
Weight (g) ^b^	2640 ± 788.9	2894 ± 762.3	2.640 ± 993.7	>0.05 ^g^
Apgar score at 1 minute ≤ 7 ^c^	4 (22.2%)	3 (42.8%)	7 (25.0%)	> 0.05 ^f^
Apgar score at 5 minutes ≤ 7 ^c^	1 (5.5%)	1 (4.7%)	2 (14.8%)	> 0.05 ^f^
Injury classification				> 0.05 ^h^
Prenatal	5 (27.7%)	10 (47.6%)	9 (31.0%)	
Perinatal	8 (44.4%)	9 (42.8%)	15 (51.7%)	
Postnatal	1 (5.5%)	1 (4.7%)	1 (3.4%)	
Unknown	4 (22.2%)	1 (4.7%)	4 (13.8%)	
Neuroimaging abnormalities ^d^	12 (66.6%)	23 (63.8%)		
Family history	1 (5.5%)	2 (9.4%)		> 0.05 ^f^
≥ 2 injuries	4 (22.2%)	6 (28.5%)	8 (27.6%)	> 0.05 ^f^
History of epilepsy	7 (38.8%)	10 (47.6%)	29 (65.5%)	> 0.08 ^f^

Abbreviations: ACTH, adrenocorticotropic hormone; CER, clinical and electroencephalographic remission.

Notes: ^a^ Data are presented as median and interquartile range values; ^b^ Data are presented as mean ± standard deviation values, n = 36; ^c^ n = 37; ^d^ n = 38; ^e^ Mann-Whitney test; ^f^ Fisher's test; ^g^ Student's *t* -test; ^h^ Pearson's Chi-squared test. Values of *p* < 0.05 indicate statistical significance.

After 30 days of therapy, 8 (21.5%) of the 37 patients with CER experienced a WS relapse. Those using a higher vigabatrin dose exhibited lower relapse rates, although no statistically significant difference was observed ( *p* = 0.09). Participant characteristics including sex, mode of delivery, gestational age, birth weight, Apgar score at 1 and 5 min, time of injury (pre-, peri-, or postnatal), 2 or more abnormal neuroimaging findings, duration of ACTH therapy, and ACTH dose did not significantly influence the relapse rates.

Adverse effects during CT were observed in 84% of the patients, including hypertension (48.7%), dyskinesia (41.0%), infections (30.7%), and neuroimaging abnormalities (23.1%). Patients who were older at the time of spasm onset had a significantly higher frequency of ACTH-related adverse effects ( *p* = 0.03). No other WS- or treatment-related factors demonstrated a significantly association with adverse effects. Nine (23.1%) patients showed vigabatrin-associated brain abnormalities on MRI (VABAM), which improved at the end of the ACTH therapy and with the discontinuation of vigabatrin. Patients with symptomatic WS had higher, although not significantly dyskinesia rates than those with unknown etiologies ( *p* = 0.06). Other WS- and treatment-related factors did not influence the occurrence of dyskinesias and VABAM.

A total of 38 patients completed the last follow-up (range 12–120 months), mostly through in-person visits (81.5%). A single patient was not found and could not be assessed. The mean age was 6 years, and 28 (73.6%) patients showed no epilepsy, including 2 patients with Lennox–Gastaut syndrome ( Figure 1 ). The main characteristics ( Table 2 ) and neuroimaging findings of the patients did not impact the progression to epilepsy or the presence/absence of epileptic seizures at the last follow-up visit. Patients who had maintained CER at the 1-year follow-up were less likely to present with progression to other epilepsy syndromes, although not significantly ( *p* = 0.08, 71.4% versus 100%), and had a significantly lower rate of epileptic seizures ( *p* = 0.03) at the last evaluation. The relapse rate was 21% at the 1-year follow-up visit. Patients with a longer delay in initiating ACTH treatment ( *p* = 0.06) or those who experienced relapse after completing CT ( *p* = 0.03) had a higher likelihood of reporting epileptic seizures at the last follow-up visit.

## DISCUSSION

Almost 75% of the patients in our study achieved CER 1 year following CT initiation. Other studies on CT in patients with WS have reported the efficacy of administering two medications simultaneously. 8 16 These studies indicate that ACTH leads to remission, on average, two days earlier than vigabatrin. 8 9 In contrast, 46.1% of our patients achieved CER by the 7th day of CT. Unlike our study, in which CT was not the initial treatment, those studies administered vigabatrin and ACTH as the first-line therapy.

O'Callaghan et al. 8 demonstrated a 72% efficacy of CT using tetracosactide or prednisolone with vigabatrin for spasm resolution on the 14th and 42nd days after treatment initiation. The efficacy decreased to 66% for complete electroclinical remission (CER). Unlike our study, theirs investigated the simultaneous administration of vigabatrin (50 mg/kg per day) and tetracosactide (0.5 mg) on alternate days in patients newly diagnosed with WS. Another study reported a similar effectiveness (60%) at the 6-month follow-up after treatment with ACTH (0.0125 mg/kg per day) and vigabatrin (50–200 mg/kg per day every 12 hours). 16

Our study displayed a significant and crescent-shaped change in the CER rate on the 7 ^th^ and 30 ^th^ days (46.1% and 94.8%, respectively; *p* < 0.001) among patients who received major doses of tetracosactide (0.5 up to 1.0 mg/kg per day) associated with vigabatrin (100–200 mg/kg per day), possibly due to the increasing effects of the tetracosactide depot and drug effect synergisms. 17 Other factors explaining the results obtained after 30 days of CT include the impact of elevated vigabatrin doses (median, 150 mg/kg per day) on ACTH therapy efficacy 18 and its synergistic interaction with the depot formulation. 19

A long time to therapy unfavorably affects the outcome; 5 6 we observed that patients with a long lag to ACTH treatment exhibited unfavorable outcomes on the 7 ^th^ day of therapy. Almost 40% of our patients had previous WS diagnoses, and most started treatment at other institutions.

### Sustained clinical and electroencephalographic remission

After 1 year of CT, most patients (76.9%) remained in CER. These findings align with previous studies, 3 13 which reported an 85% treatment efficacy at 18 months for CT with vigabatrin and ACTH or prednisolone in patients with a recent WS diagnosis. Our results are consistent with the high WS resolution rates reported by these authors, who defined the absence of epileptic spasms as their primary outcome measure. Additionally, before the CT, nearly 30% of our study population had failed to respond to monotherapy with vigabatrin and other ASMs, which could influence drug response.

The timing of ACTH therapy, vigabatrin dose, age of spasm onset, presence of two or more etiologies, did not impact the sustained efficacy. However, a five times higher likelihood toward unfavorable outcomes was noted in female patients, possibly attributable to the absence of cryptogenic etiology in this subgroup and the small sample size. Knupp et al. 7 and O'Callaghan et al. 8 did not report any influence of sex on the treatment response.

The etiology of WS can also influence outcomes. Osborne et al. demonstrated good efficacy of CT in patients with vascular brain (72%) and perinatal (66%) injuries. 20 In our study, premature patients had a significantly higher probability of sustained CER than term patients. Souza et al. reported a CER of 73% after CT in patients with trisomy 21. 21

Examining the long-term relapse rates is important for assessing whether the treatment response is maintained. 3 Relapse occurs in 50% of patients during the 1st year of follow-up after initial CER. 16 22 In the continuation of the International Collaborative Infantile Spasms Study, 7% of relapses occurred following CT (vigabatrin and prednisolone or ACTH) in patients newly diagnosed with WS. 13 In contrast, we observed 3 times more relapses than those observed in that study at the 1-year follow-up visit. The broad symptomatic presentation (87%) and vigabatrin withdrawal after the 6th month can play a role in relapses. Although prolonged ACTH therapy (more than 1 month) has been reported to reduce the relapse rate, 6 this effect was not observed in our study (0.5 versus 1.0 mg/kg per day of ACTH).

### Tolerability

In line with previous findings, 3 8 22 23 24 most patients (84.6%) in our study, experienced adverse effects during CT. Notably, patients receiving double doses of ACTH showed a tendency toward developing adverse events. Structural changes, such as VABAM, can also occur, mainly in young patients and those receiving high doses of vigabatrin. 25 26 27 However, in our study, VABAM was not associated with age, time of spasm onset, and drug dose. Severe adverse events are less frequently observed during combined drug treatment. 3 Similar to results of previous studies, no fatal adverse effects were observed in our study. Nevertheless, two patients developed pneumonia, leading to the discontinuation of ACTH therapy.

### Progression to other forms of epilepsy

Most studies on CT for WS had short follow-up durations, 3 7 8 16 which influence the availability of epilepsy progression data. In our study, 68% of patients had an epilepsy diagnosis at a median of 21.5 months, compared with 30% at 18 months in a large-scale prospective study on CT use conducted by O'Callaghan et al. in 2018. 13 This difference can be explained by the difference in the proportions of patients with symptomatic WS between our (87%) and their (58%) study, as this is an important factor for epilepsy development in patients with WS. Additionally, O'Callaghan et al. included patients with a new WS diagnosis, and a considerable proportion of them had favorable prognostic factors. 13

O'Callaghan et al. also showed that higher WS remission rates following CT do not decrease progression to epilepsy. 13 In contrast, patients with sustained CER at the final evaluation (median, 21.5 months) in our study were significantly less likely to show progression to epilepsy. Additionally, they had a significantly lower risk of reporting epileptic seizures at the final follow-up visit. Almost 50% of patients with WS in the previous study maintained their ASM use at 18 months of follow-up, 13 whereas 73.6% of the patients in our study used ASMs at the time of the final evaluation. The sample selection method, prior treatment with conventional ASMs, and delayed treatment in the present study may have played a role in this discrepancy. 9

### Limitations

Neurodevelopmental status was not objectively assessed in either of the study phases, limiting the inferences that can be drawn from our results. Moreover, the sample size, and lack of a control group for comparing drugs other than vigabatrin used for CT with ACTH were limitations in our study.

In conclusion, the current study demonstrates a favorable response to a second-line therapy with sequential ACTH and vigabatrin as a combined treatment. Also, the study showed that higher CER rate was associated with a lower progression to epilepsy. Further prospective research is needed to refine outpatient protocols, assess the role of oral corticosteroids, and validate these findings across all WS patients.
